# Angelman Syndrome Protein UBE3A Interacts with Primary Microcephaly Protein ASPM, Localizes to Centrosomes and Regulates Chromosome Segregation

**DOI:** 10.1371/journal.pone.0020397

**Published:** 2011-05-25

**Authors:** Pooja Singhmar, Arun Kumar

**Affiliations:** Department of Molecular Reproduction, Development and Genetics, Indian Institute of Science, Bangalore, Karnataka, India; UT Southwestern Medical Center, United States of America

## Abstract

Many proteins associated with the phenotype microcephaly have been localized to the centrosome or linked to it functionally. All the seven autosomal recessive primary microcephaly (MCPH) proteins localize at the centrosome. Microcephalic osteodysplastic primordial dwarfism type II protein PCNT and Seckel syndrome (also characterized by severe microcephaly) protein ATR are also centrosomal proteins. All of the above findings show the importance of centrosomal proteins as the key players in neurogenesis and brain development. However, the exact mechanism as to how the loss-of-function of these proteins leads to microcephaly remains to be elucidated. To gain insight into the function of the most commonly mutated MCPH gene *ASPM*, we used the yeast two-hybrid technique to screen a human fetal brain cDNA library with an ASPM bait. The analysis identified Angelman syndrome gene product UBE3A as an ASPM interactor. Like ASPM, UBE3A also localizes to the centrosome. The identification of UBE3A as an ASPM interactor is not surprising as more than 80% of Angelman syndrome patients have microcephaly. However, unlike in MCPH, microcephaly is postnatal in Angelman syndrome patients. Our results show that UBE3A is a cell cycle regulated protein and its level peaks in mitosis. The shRNA knockdown of UBE3A in HEK293 cells led to many mitotic abnormalities including chromosome missegregation, abnormal cytokinesis and apoptosis. Thus our study links Angelman syndrome protein UBE3A to ASPM, centrosome and mitosis for the first time. We suggest that a defective chromosome segregation mechanism is responsible for the development of microcephaly in Angelman syndrome.

## Introduction

Centrosomal proteins function in tango with cell cycle proteins and the loss-of-function of these proteins leads to cell cycle misregulation [1]. Mutations in many centrosomal protein encoding genes have been linked to microcephaly, indicating the importance of centrosomes in the expansion of neuronal progenitor cells. Microcephaly is a neurological disorder characterized by a reduced brain volume and head circumference (HC) which is three standard deviations below the average HC for an individual's age and sex.

Autosomal recessive primary microcephaly (MCPH; OMIM 251200) is a subtype defined by congenital microcephaly and associated mental retardation (MR). It is genetically heterogeneous with seven known loci and the genes for all seven loci have been identified: MCPH1-*MCPH1*, MCPH2-*WDR62*, MCPH3-*CDK5RAP2*, MCPH4-*CEP152*, MCPH5-*ASPM*, MCPH6-*CENPJ*, and MCPH7-*STIL* [2], [3], [4], [5], [6], [7], [8].

The most common cause of MCPH is mutations in the *ASPM* (abnormal spindle-like, microcephaly-associated protein) gene [3], [9], [10], [11], [12]. *ASPM* is the human orthologue of the *Drosophila melanogaster asp* (abnormal spindles) gene. It encodes for a 3,477 amino acids protein and is widely expressed in many human fetal and adult organs including brain, kidney, muscle and lung [13]. Using RNAi knockdown approach, Fish and colleagues demonstrated that the loss of *Aspm* in mouse neuroepithelium alters the orientation of cleavage plane in the progenitor cells, resulting in an increase in asymmetric divisions and reduction in the neuronal progenitor pool [14].

Bioinformatic analysis has predicted the following domains and motifs in ASPM: an N-terminal microtubule binding domain, two calponin homology domains, 81 IQ (isoleucine-glutamine) repeat motifs, and a C-terminal region with a conserved armadillo-like repeat domain [12]. A recent *C. elegans* study demonstrated that the IQ motifs in ASPM-1 associate with calmodulin to affect ASPM-1's centrosomal localization [15]. Apart from IQ motifs, the rest of the domains remain to be characterized functionally. Interestingly, the loss of the last 149 amino acids from the C-terminal region of ASPM is sufficient to cause MCPH [9], indicating that it is indispensable for the function of ASPM and directly involved in neurogenesis. The purpose of this study was to investigate the novel protein interactions mediated by the C-terminal region of ASPM.

## Results

### Yeast two-hybrid (Y2H) analysis with a C-terminal region of ASPM

**Figure 1:**
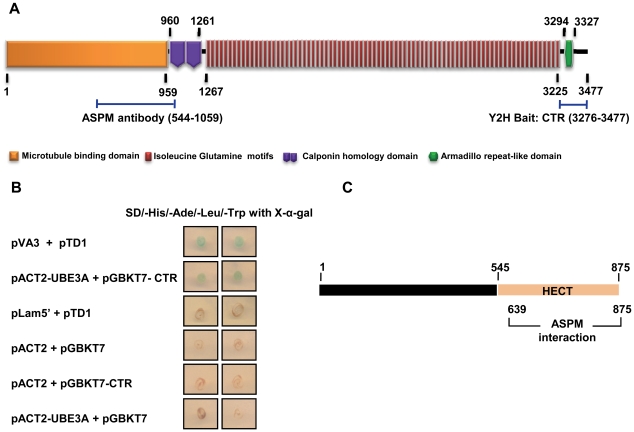
Identification of interaction between ASPM and UBE3A by Y2H analysis. (A) Schematic representation of putative domains and motifs in ASPM. A C–terminal region of ASPM, named CTR (amino acids 3,276–3,477) used as a bait in Y2H analysis is shown. An N-terminal region (amino acids 544–1,059) encompassing the predicted microtubule binding domain and a part of calponin homology domain used for antibody generation is also shown. (B) Y2H screening of transformants by nutritional selection and X-α-gal plate assay. Note growth and blue color for transformants harboring pVA3+pTD1 (positive control) and pACT2-UBE3A+pGBKT7-CTR. As expected, no growth and blue color were observed for transformants harboring pLam5′+pTD1, pACT2+pGBKT7, pACT2+pGBKT7-CTR and pACT2-UBE3A+pGBKT7. (C) Schematic representation of UBE3A protein. The ASPM interacting region of UBE3A (amino acids 639–875) is located within the HECT domain (amino acids 545 to 875). The numbers represent amino acid positions.

To find novel interacting partners for the C-terminal region (CTR) of ASPM, we cloned this region corresponding to amino acids 3,276–3,477 in the Y2H DNA binding domain vector pGBKT7 (Figure 1A). The clone (pGBKT7-CTR) was subsequently used as a bait to screen a human fetal brain cDNA library cloned in the Y2H activation domain vector pACT2. The screen identified eight transformants which were further tested for growth and blue color on plates with quadruple dropout medium (SD/-His/-Ade/-Leu/-Trp) and X-α-gal. DNA sequence analysis of the pACT2 plasmid from one of the transformants showed that it harbours a 714 bp long fragment of UBE3A (ubiquitin protein ligase E3A) corresponding to amino acids 639–875. The 714 bp fragment of UBE3A was recloned in pACT2 (pACT2-UBE3A) and co-transformed with the bait pGBKT7-CTR in yeast cells. The transformant was re-assessed by nutritional selection and X-α-gal plate assay as described above. The growth of the transformant and blue color suggested that ASPM interacts with UBE3A (Figure 1B). The region of UBE3A from amino acids 639–875 overlaps with the HECT domain (homologous to the E6AP carboxyl terminus) of UBE3A which is known to be involved in ubiquitination of its target proteins (Figure 1C).

### ASPM interacts with UBE3A *in vivo*

**Figure 2:**
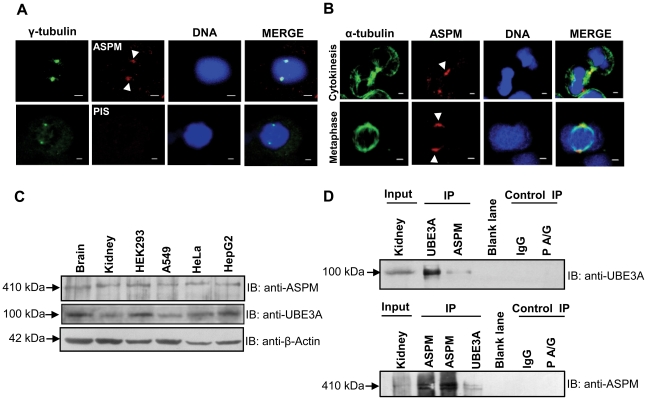
Localization of ASPM and interaction of ASPM with UBE3A *in vivo*. (A) Indirect immunofluorescence of HEK293 cells stained with anti-γ-tubulin and raised anti-ASPM antibodies. Upper panel: Note ASPM (red) colocalizes with the centrosomal marker γ-tubulin (green). Lower panel: Pre-immune serum (PIS/normal IgG) does not show any signal. Scale bar = 2 µm. (B) Immunofluorescence images of HEK293 cells stained with anti-α-tubulin and raised anti-ASPM antibodies. Upper panel: note ASPM localizes at midbody during cytokinesis (arrow heads). Lower panel: note ASPM localizes at spindle poles during metaphase (arrow heads). Scale bar = 2 µm. (C) Expression profile of ASPM and UBE3A by Western blot analysis using lysates from human fetal brain, human fetal kidney, HEK293, A549, HeLa and HepG2. Anti-ASPM and anti-UBE3A (anti-UBE3A-sc-8926) antibodies recognize 410 kDa and 100 kDa bands, respectively. Note the ubiquitous expression of both proteins. (D) Co-immunoprecipitation of ASPM and UBE3A in human fetal kidney tissue. Upper panel: immunoprecipitation of ASPM co-precipitates UBE3A (100 kDa). Lower panel: immunoprecipitation of UBE3A co-precipitates ASPM (410 kDa). Input lane represents tissue lysate used in pulldown. None of these proteins coprecipitated with either normal IgG or PA/G beads. Abbreviations: IB, immunoblotting; and, IP, immunoprecipitation.

To confirm the interaction of ASPM with UBE3A using co-immunoprecipitation *in vivo*, we first raised a rabbit polyclonal antibody against the N-terminal region of ASPM (Figure 1A), as a commercial antibody was unavailable when we started this experiment. The specificity of the ASPM antibody was verified by Western blotting (Figure S1) and immunofluorescence (Figure 2A and B). The raised anti-ASPM antibody recognized the 56 kDa immunogen (Figure S1A). As expected Western blot analysis with human fetal kidney lysate showed a 410 kDa band corresponding to a full-length ASPM protein (Figure S1B). The same result was obtained with a commercial ASPM antibody when it became available (Figure S1B). Here after, we have used the anti-ASPM antibody raised in our laboratory for all the experiments.

Immunofluorescence staining of HEK293 cells with anti-ASPM antibody showed immunoreactivity at the centrosome as reported earlier [13] (Figure 2A). The specific staining of centrosomes by anti-ASPM antibody was confirmed by its colocalization with γ-tubulin, a centrosomal marker (Figure 2A, upper panel). The specificity of the anti-ASPM antibody was further confirmed by absence of centrosomal staining with rabbit pre-immune serum (Figure 2A, lower panel). As reported in the literature, the anti-ASPM antibody was found to stain midbody during cytokinesis in HEK293 cells [16] (Figure 2B, upper panel). ASPM localization was also seen at MTOC (microtubule organizing center) in mitotic cells as it coimmunostained with α-tubulin (Figure 2B, lower panel). Further, a 410 kDa ASPM band was detected in lysates from human fetal brain and four cell lines (viz., HEK293, A549, HeLa and HepG2) by Western blotting (Figure 2C). In the same panel of lysates, goat anti-UBE3A-sc-8926 antibody detected the expected 100 kDa band (Figure 2C). The physical interaction between ASPM and UBE3A was confirmed *in vivo* using human fetal kidney lysate. Due to the limited availability of human fetal brain tissue, we used the fetal kidney tissue for co-immunopreciptation studies as it was readily available. Immunoprecipitation using anti-ASPM antibody followed by immunoblot analysis using anti-UBE3A-sc-8926 antibody detected a 100 kDa band corresponding to the expected size of UBE3A (Figure 2D). Similarly, immunoprecipitation with anti-UBE3A antibody pulled down ASPM (Figure 2D). Pre-immune serum (PIS/IgG) and protein A/G beads (P A/G) did not pull down either of the proteins (Figure 2D).

### UBE3A localizes to the centrosome throughout mitosis

**Figure 3:**
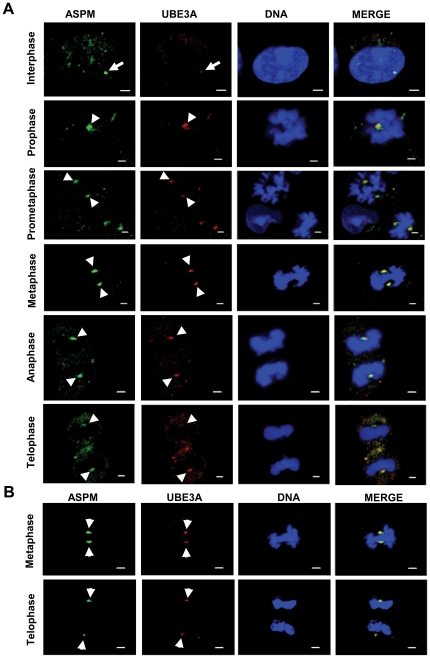
UBE3A colocalizes with ASPM at the centrosome. (A) Indirect immunofluorescence of HEK293 cells at interphase and different phases of mitosis stained with antibodies against ASPM and UBE3A (anti-UBE3A-sc-8926). Note colocalization of UBE3A with ASPM at the centrosome throughout mitosis (arrowheads). Note weak centrosomal staining of UBE3A in an interphase cell (arrow). (B) Indirect immunofluorescence of A549 cells stained with antibodies against UBE3A (anti-UBE3A-sc-8926) and ASPM at metaphase and telophase. Note colocalization of UBE3A with ASPM at the centrosome (arrowheads). Scale bar = 2 µm.

**Figure 4:**
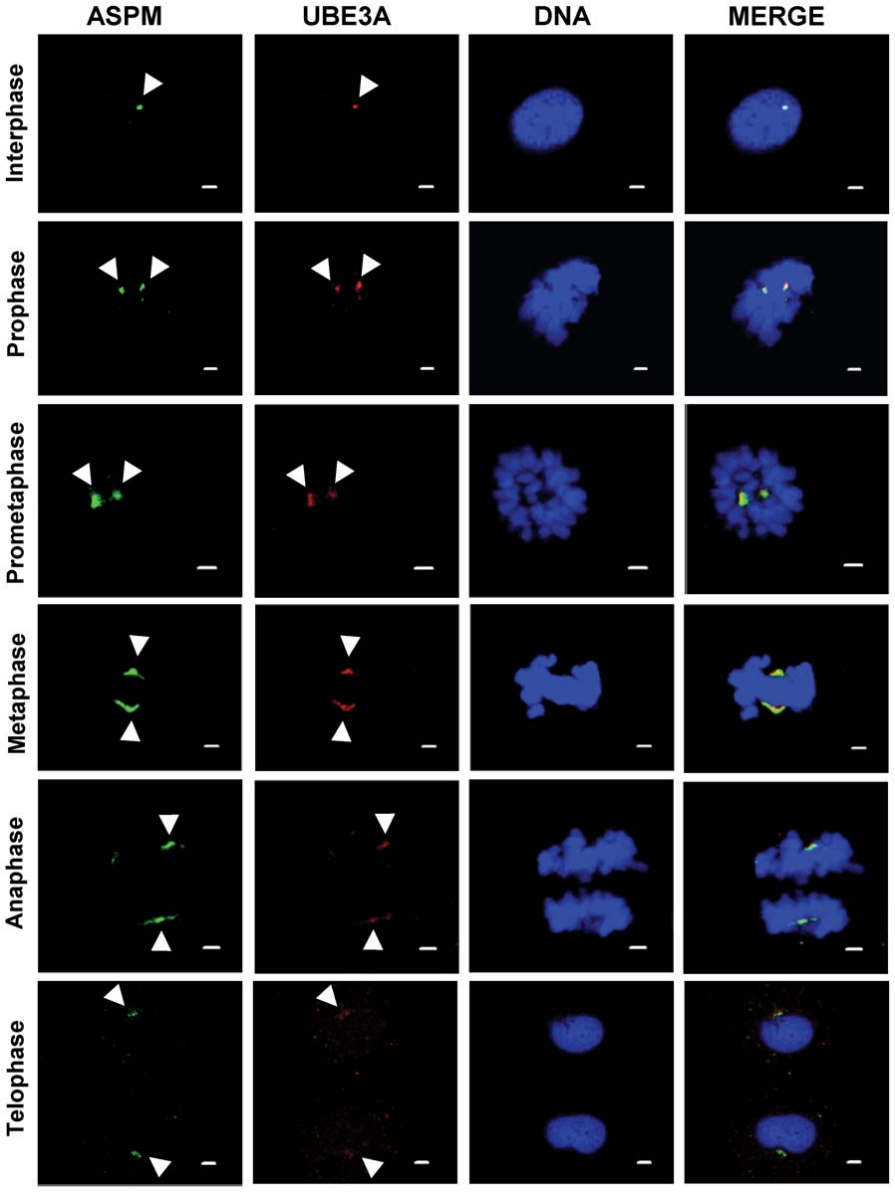
Confirmation of colocalization of UBE3A with ASPM at the centrosome using a different UBE3A antibody. Indirect immunofluorescence of HEK293 cells at interphase and different phases of mitosis stained with antibodies against ASPM and UBE3A (anti-UBE3A-sc-12380). Note anti-UBE3A-sc-12380 antibody (arrowheads) also shows a centrosomal staining pattern as observed with anti-UBE3A-sc-8926 antibody. Scale bar = 2 µm.

An analysis of UBE3A immunofluorescence staining with anti-UBE3A-sc-8926 antibody in HEK293 cells by confocal microscopy revealed that it stains the centrosome throughout the mitotic progression from prophase to telophase and colocalizes with ASPM (Figure 3A). Identical results were obtained in A549 cell line (Figure 3B). In interphase cells, UBE3A staining observed at the centrosome was weak (Figure 3A). A similar result was obtained using a different anti-UBE3A antibody (anti-UBE3A-sc-12380), confirming the centrosomal localization of UBE3A (Figure 4). Henceforth, we have used the anti-UBE3A-sc-8926 antibody for all the experiments.

### UBE3A is a cell cycle dependent protein and its overexpression does not degrade ASPM

**Figure 5:**
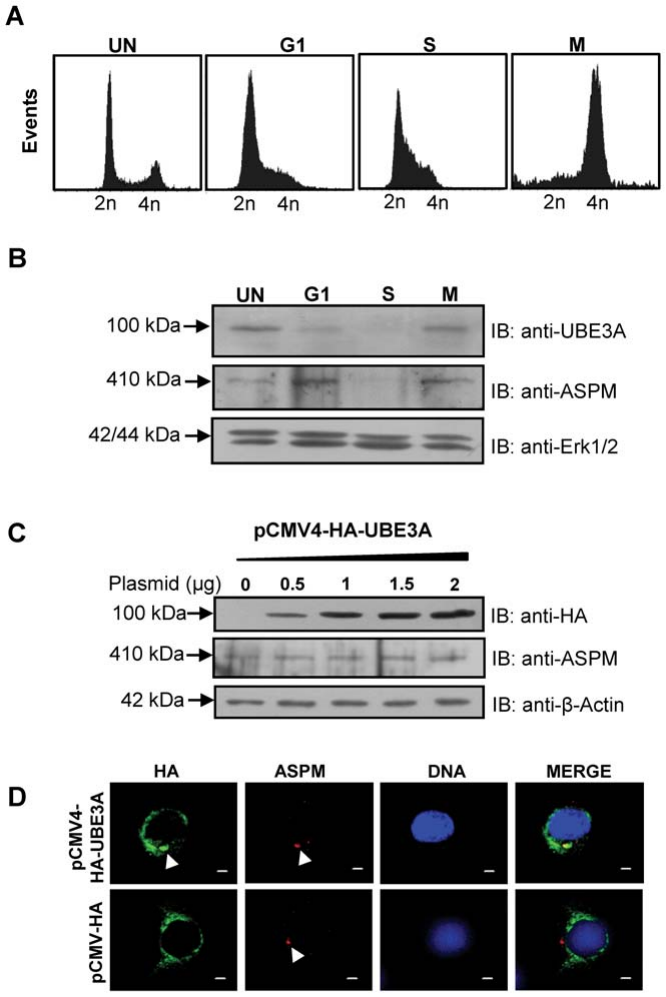
UBE3A is a cell cycle dependent protein and it fails to degrade ASPM. (A) Flow cytometric analysis of cells to confirm synchronization in different cell cycle phases. Note the cells in G1 phase show a 2n peak, cells in M phase with a 4n peak and cells in S phase fall in between 2n-4n peaks, suggesting cell cycle arrest at these phases. UN: unsynchronized cells. (B) HEK293 cells synchronized at different cell cycle phases were analyzed for UBE3A and ASPM expression by Western blot analysis. Note the expression of UBE3A was found to peak in M phase as compared to its levels in G1 and S phases. The level of ASPM peaks during G1 and M phases. Erk1/2 was used a loading control. The level of Erk1/2 is known to remain unaffected during different phases of cell cycle. (C) Western blot analysis of lysates from HEK293 cells transfected with an increasing concentration of the pCMV4-HA-UBE3A construct. Note UBE3A overexpression fails to degrade ASPM. Overexpression of UBE3A was determined by probing the blot with anti-HA antibody. β-Actin was used as a loading control. (D) Immunofluorescence analysis of HEK293 cells overexpressing the pCMV4-HA-UBE3A construct. UBE3A and ASPM staining were examined by anti-HA and anti-ASPM antibodies respectively. Note overexpression of UBE3A does not alter ASPM localization or protein level (compare ASPM signals in upper and lower panels). Also note colocalization of UBE3A with ASPM at the centrosome (upper panel). Cells transfected with the pCMV-HA vector were also stained with anti-HA antibody as a control (lower panel).

We investigated the role of UBE3A in cell cycle further by studying its cell cycle dependence. For this, we synchronized HEK293 cells at three different stages- G1/S boundary, M phase and S phase. The cells were stained with propidium iodide (PI) for DNA content and analyzed by Flow cytometry to check for the efficiency of synchronization (Figure 5A). Western blot analysis of synchronous cells revealed that UBE3A levels fluctuate across the cell cycle phases (Figure 5B). UBE3A was found to be maximally expressed in M phase (Figure 5B). Whereas a minimum expression of UBE3A was seen in S phase (Figure 5B). We observed that the expression level of ASPM in S phase corroborates with UBE3A (Figure 5B). Taken together, above observations imply that UBE3A is under cell cycle regulation and its high expression level in mitosis may be playing an important function.

To determine if the level of ASPM protein is regulated by UBE3A, we overexpressed UBE3A in HEK293 cells by transient transfection of the pCMV4-HA-UBE3A construct and the cell lysates were examined for ASPM levels. ASPM levels were found to be unaffected (Figure 5C), indicating that ASPM is not degraded by a UBE3A-dependent proteasomal pathway or the degradation may be under spatial-temporal control. Further, we wanted to check if overexpression of UBE3A affects the localization or accumulation of ASPM at centrosomes. Immunofluorescence analysis of UBE3A overexpressing HEK293 cells revealed that UBE3A does not affect either the ASPM localization or its protein levels at the centrosome (Figure 5D). The centrosomal localization of UBE3A was further confirmed as HA-tagged UBE3A was found to be colocalized with ASPM to the centrosome (Figure 5D).

### UBE3A depleted cells show chromosome segregation defects and abnormal spindles

**Figure 6:**
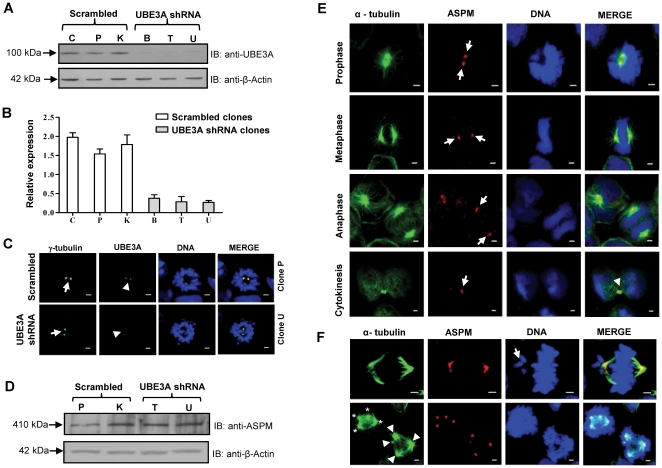
Generation of stable UBE3A shRNA knockdown clones and analysis of mitotic stages. (A) HEK293 cells transfected with a HuSH 29mer shRNA construct against UBE3A and puromycin resistant clones (B, T and U) were examined for knockdown by Western blot analysis. As a control, clones (C, P and K) transfected with scrambled shRNA were also generated. Note reduced expression of UBE3A in clones B, T and U as compared to scrambled clones C, P and K. (B) Densitometric analysis of blot in panel A. UBE3A knockdown clones T and U showed approximately 80% knockdown as compared to the scrambled clone P and K. Bars represent average values from densitometric analysis of the bands obtained in three separate experiments. (C) Representative images of scrambled and UBE3A shRNA knockdown prometaphase cells showing UBE3A staining. Note a reduced UBE3A signal (arrowheads) at the centrosome in an UBE3A shRNA knockdown cell as compared to a scrambled cell. γ-tubulin was used for centrosomal staining (arrows). (D) Western blot analysis of lysates from scrambled clones P and K, and UBE3A knockdown clones T and U with anti-ASPM antibody. Note that the ASPM protein level remains unaffected in UBE3A knockdown clones as compared to scrambled clones. β-Actin was used as a loading control. (E) Representative images of mitotic cells at different stages from scrambled clone P showing normal chromosome alignment and mitotic spindles. Arrows point to ASPM staining and arrowhead marks the midbody. Scale = 2 µm. (F) Cells from UBE3A knockdown clone T showing misalignment of chromosomes and multipolarity at metaphase. Note an arrow points to misaligned chromosomes which lie outside the spindle poles (upper panel). Note in two multipolar cells (lower panel), the different poles are marked with arrowheads for lower right cell and marked with “*” for upper left cell. Scale = 2 µm.

To ascertain the functional significance of an increased level of UBE3A in M phase (Figure 5B) and its interaction with ASPM, we depleted the expression of UBE3A in HEK293 cells with a shRNA (short hairpin RNA) construct and generated stable cell lines. Western blot analysis of three stable clones (B, T and U) showed more than 80% knockdown in UBE3A expression and two clones (T and U) were further used in analysis (Figure 6A and B). As a negative control, two (P and K) of three scrambled clones were also used (Figure 6A and B). Immunofluorescence analysis of UBE3A shRNA knockdown clones revealed a reduced staining of UBE3A at the centrosome as compared to scrambled clones (Figure 6C). No significant difference was found in the protein level of ASPM in UBE3A depleted cells as compared to scrambled cells (Figure 6D), suggesting that the interaction of UBE3A with ASPM has no role in regulating the protein level of ASPM.

**Figure 7:**
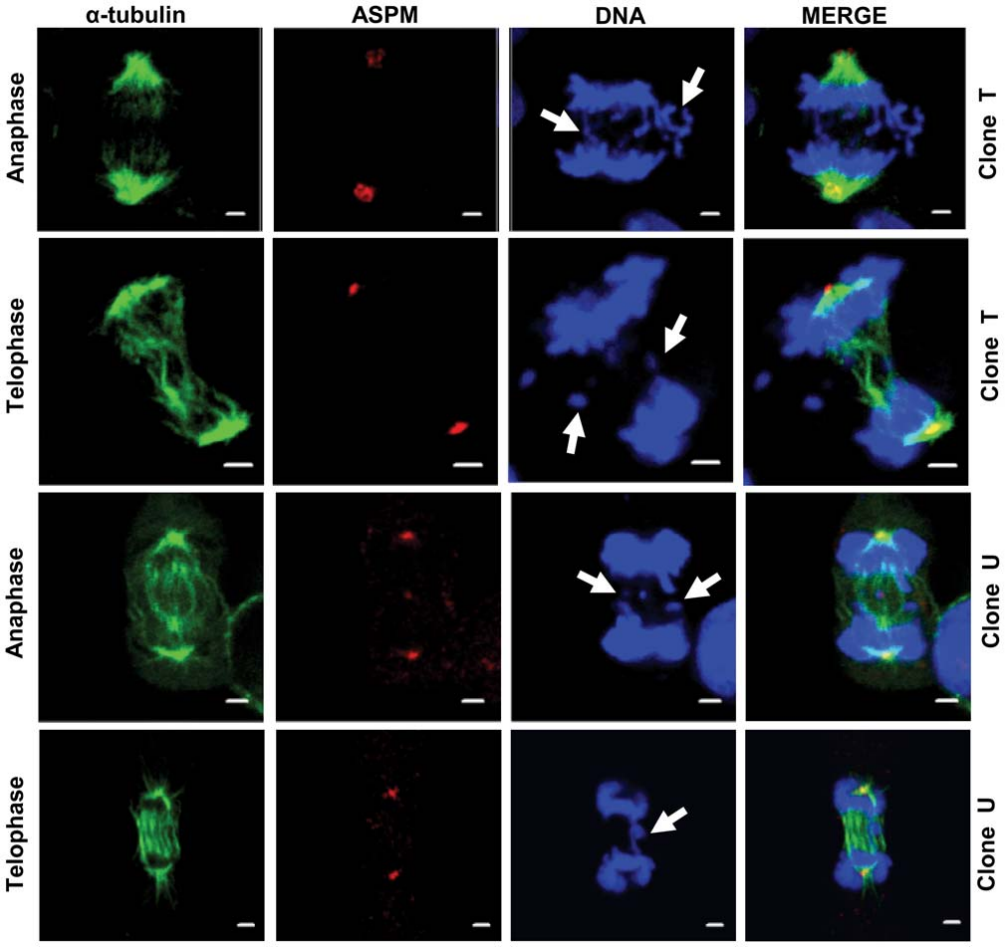
Chromosome segregation defects associated with abnormal spindles in UBE3A shRNA knockdown clones. Examples of cells attempting to divide with missegregated (clone T, anaphase) and lagging chromosomes (clone U, telophase) (arrows). Also note cells with chromosomes hanging at the equatorial plane (clone U, anaphase and clone T, telophase) (arrows). Note the cells have disorganized mitotic spindles (all four panels) and bundling of microtubules at the central spindle (clone U, telophase) and at MTOC (clone T, anaphase). Scale = 2 µm.

Confocal microscopy analysis of UBE3A knockdown cells with triple staining for ASPM (red), α-tubulin (green) and DNA (blue) showed mitotic defects (Table 1). We examined the cells at different stages of mitosis for defects in unsynchronized cultures of two UBE3A knockdown clones (clone T, n = 152 and clone U, n = 162) and two control scrambled shRNA clones (clone P, n = 153 and clone K, n = 157). We scored the cells for the following abnormalities: prometaphase/metaphase cells for misaligned chromosomes and multipolarity (Figure 6E and F) and anaphase/telophase cells for missegregation/lag of chromosomes (Figure 7). ASPM remained at the centrosome in UB3EA depleted cells indicating that its centrosomal localization is UBE3A independent (Figures 6F and 7). The cells in prophase displayed no abnormalities and a relatively low frequency of defects was found in metaphase (Table 1). At anaphase/telophase stage, defects were more evident as cells showed an increased frequency of lagging chromosomes and missegregation (Figure 7; Table 1). Chromosome missegregation/lag was observed in 38.71% and 45.10% of clone T and clone U anaphase/telophase cells respectively as compared to 10.17% in scrambled clone P and 8.16% in scrambled clone K cells (Table 1). Interestingly, we observed that most of the UBE3A shRNA knockdown cells displaying chromosome missegregation showed abnormal spindles (Figure 7).

**Table 1:** Quantitation of mitotic abnormalities in scrambled and UBE3A shRNA knockdown cells.

		Scrambled clone P (n = 153)	Scrambled clone K (n = 157)	UBE3A shRNA clone T (n = 152)	UBE3A shRNA clone U (n = 162)
**Prophase**		0/9 = 0%	0/13 = 0%	0/13 = 0%	0/12 = 0%
**Prometaphase/Metaphase**	**Misaligned chromosomes**	4/85 = 4.71%	1/95 = 1.05%	7/77 = 9.09%	6/99 = 6.06%
	**Multipolarity**	12/85 = 14.12%	12/95 = 12.63%	17/77 = 22. 08%	19/99 = 19.19%
**Anaphase/Telophase**	**Missegregation/Lag**	6/59 = 10.17%	4/49 = 8.16%	24/62 = 38.71%	23/51 = 45.10%

Abbreviation: n = total number of mitotic cells observed.

### UBE3A knockdown leads to abnormal cytokinesis and apoptosis

**Figure 8:**
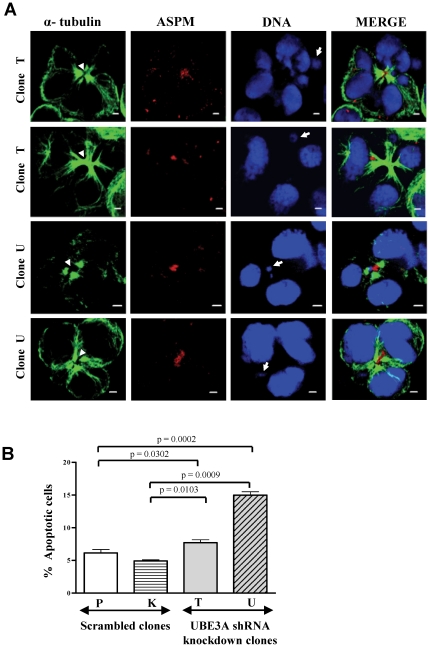
Abnormal cytokinesis and apoptosis in UBE3A knockdown cells. (A) Note cells undergoing cytokinesis with elongated nuclear morphology giving rise to abnormal number of nuclei. An extended midbody (arrowheads) and micronuclei (arrows) can be seen in all the panels. ASPM was found to be diffusely present at the midbody. Scale = 2 µm. (B) Quantitation of apoptosis by *in vivo* detection of caspase-3 activity. Note UBE3A shRNA clones showed a significant increase in apoptotic cells as compared to scrambled clones. Cells were analyzed by flow cytometry using FL-1 channel (10,000 cells were measured for each sample). Mean ± SEM values for the samples is as follows: scrambled clone P = 6.150±0.2972, scrambled clone K = 4.920±0.0986, UBE3AshRNA clone T = 7.700±0.2663, and UBE3AshRNA clone U = 14.99±0.2929. Data are representative of three independent experiments. Unpaired Student's t-test was used to determine the significance of difference between scrambled and UBE3A knockdown clones.

We next examined if chromosome missegregation/lag in UBE3A depleted cells is leading to cytokinesis failure and micronuclei formation. Cytokinesis failure is characterized by an extended nuclear morphology reminiscent of missegregated chromosomes and an elongated midbody [17]. We could notice many of these phenotypes in UBE3A depleted cells (Figure 8A). The scrambled clones P and K displayed normal cytokinesis (Figure 6E). These defects appear when the lagging chromosomes are pushed by the cleavage furrow into either one of the two daughter cells, leading to micronuclei formation and abnormal cytokinesis (Figure 8A). We were unable to score for cytokinesis abnormalities due to low percentage of these cells. This may be due to rescue of the abnormal phenotype or cell death. Malfunctioning of chromosome segregation can generate aneuploid cells and these cells are usually inviable. With this reasoning, we examined the caspase-3 activation in cells to monitor apoptosis. We noticed a significantly higher percentage of caspase-3 positive cells in UBE3A depleted cells as compared to scrambled cells (Figure 8B). For example, the percent of apoptotic cells in UBE3A depleted clone U was 3-fold higher as compared to scrambled clone K (Figure 8B).

## Discussion

The C-terminal region of ASPM is a prerequisite for its function, as its loss is enough to cause MCPH [9]. The precise functional role of this indispensable region of ASPM and its interactions with other proteins has been lacking. Given this scenario, we screened a human fetal brain library with an ASPM C-terminus bait and identified Angelman syndrome (AS) gene product UBE3A as an interacting partner. This is the second reported interaction of ASPM, apart from citron kinase [16]. Mutation in *CITK* is known to cause severe microcephaly in rats and mice [18]. The identification of UBE3A as an ASPM interactor is not unexpected as more than 80% of AS patients have microcephaly [19]. However, unlike in MCPH where microcephaly is congenital, it develops postnatally in AS patients usually by the age of two years [19]. Further, AS patients have other clinical features (e.g., ataxia, a characteristic electroencephalogram, happy- puppet-like gait and inappropriate laughter) that are not present in MCPH patients. Therefore, the molecular mechanisms involved in the development of microcephaly in AS could be different than in MCPH [20], [21]. It is interesting to note that overall reduction in brain size (microcephaly) has been reported in a mouse model of AS [22]. These studies underline the role of UBE3A in determining brain size. Like ASPM, Ube3a is also expressed in cerebral cortex [23]. Till date, studies indicate both cytoplasmic and nuclear localization of UBE3A [23]. We have for the first time demonstrated a centrosomal localization of UBE3A (Figure 3–4). It is surprising that UBE3A's centrosomal localization has gone unnoticed so far. Therefore, our study opens a whole new perspective on UBE3A function. The interaction of UBE3A with ASPM and its centrosomal localization reveal its new subcellular location and function in mitosis.

Although all the MCPH proteins localize at the centrosome, intriguingly their localization is cell cycle dependent [4], [6], [7], [13], [24], [25]. For example, MCPH1, WDR62, CDK5RAP2, ASPM, and CENPJ are all localized at the centrosome throughout the mitosis [4], [7], [13], [16], [24]. However, only MCPH1, CDK5RAP2, ASPM, and CENPJ are found at the centrosome in interphase cells [4], [13], [24]. Interestingly, STIL is associated with spindle poles only in metaphase cells [25]. Three of the MCPH proteins- CDK5RAP2, ASPM and CENPJ- also show accumulation at the midbody during cytokinesis [16]. The centrosomal localization of UBE3A was found to be similar to WDR62, as it stains centrosomes throughout mitosis but its centrosomal localization in interphase cells is not very prominent (Figure 3A).

To investigate the role of mitosis-dependent accumulation of UBE3A, we depleted its levels in HEK293 cells by shRNA (Figure 6A and B). With the loss of protein, we suspected the essential events that require UBE3A function to be affected. To address this, we scored for mitotic abnormalities in UBE3A depleted cells (Table 1). Our analysis identified a definitive role of UBE3A in chromosome segregation. We found a 4- to 5- fold increase in the frequency of anaphase/telophase cells with missegregated chromosomes in UBE3A knockdown clones as compared to scrambled cells (Table 1; Figure 7). And as a reminiscent phenotype of chromosome missegregation, we observed abnormal cytokinesis and the presence of micronuclei in UBE3A knockdown cells (Figure 8A). A defective chromosome segregation process generates aneuploid cells that are usually inviable [26]. As anticipated, we observed a higher percentage of apoptotic cells in UBE3A knockdown clones as compared to control clones (Figure 8B). We hypothesize that if a similar mechanism exists in the neuronal cells, the loss of UBE3A function would cause increased cell death and a reduced neural progenitor pool leading to microcephaly. It is important to note that the present study is done in an embryonic kidney cell line and future experiments need to be performed to prove that a similar mechanism exists in neuronal progenitor cells. Interestingly, an extensive apoptosis has been found in the neural folds of MCPH7 gene *STIL* null mice embryos [27].

Defective chromosome segregation has been reported in many studies associated with microcephaly-related proteins. For example, depletion of MCPH1 leads to lagging chromosomes and delayed cytokinesis in U2OS (osteosarcoma) cells [28]. However, unlike in UBE3A depleted cells, the lagging chromosomes in MCPH1 depleted cells are observed at metaphase and not at anaphase [28]. Inhibition of CDK5RAP2 expression by siRNA in HeLa cells also leads to increased chromosome misalignment and lagging chromosomes along with reduced expression of different checkpoint proteins like BUBR1 and MAD2 [29]. Moreover, Lizarraga et al. have recently implicated a *Cdk5rap2* mutation in Hertwig's anemia (*an*) mice [30]. They found an inherent predisposition to chromosomal aneuploidy in *Cdk5rap2 an/an* primary cells. Chromosome segregation defects have also been reported in *asterless* (CEP152 *Drosophila* orthologue) mutants and *Drosophila dSas-4* (CENPJ *Drosophila* orthologue) knockouts in spermatocytes during male meiosis [31], [32]. Furthermore, studies by Gonzalez et al. have shown that mutations in *Drosophila asp* cause non-disjunction in embryos and arrest of neuroblasts at metaphase [33]. RNAi knockdown of *C.elegans aspm-1* causes defects in chromosome segregation in meiosis I and II [15]. Examination of primary fibroblasts from microcephalic osteodysplastic primordial dwarfism type II patients has also provided evidence that the lack of PCNT results in disorganized mitotic spindles, together with mosaic variegated aneuploidy and premature sister chromatid separation [34]. All the above studies show chromosome missegregation as a common phenotype in cells with loss-of-function of MCPH and other microcephaly-related proteins. It emphasizes the need for an intact chromosome segregation machinery for the maintenance of an optimal neural progenitor pool for normal brain development.

UBE3A belongs to the HECT-E3 ubiquitin ligase family of enzymes [35]. Although the physiological functions of most HECT-E3s are not well understood, it is becoming clear that HECT-E3s play an important role in human diseases. HECT-E3s have been linked to AS (UBE3A), Liddle's syndrome (Nedd4-I), tuberous sclerosis complex (HERCI) and cancer (Smurf2, UBE3A and EDD), suggesting the functional spectrum of these proteins [36]. Several lines of our data confirmed that ASPM interacts with UBE3A, though a precise functional link between the two could not be ascertained. Overexpresion of UBE3A in cells can mediate degradation of its target proteins. For example, Yang et al. [37] found that overexpression of UBE3A in HeLa cells leads to degradation of its target protein trihydrophobin 1 (TH1). Shimoji et al. [38] demonstrated that overexpression of UBE3A decreased the level of annexin A1 protein in HEK 293T cells. In contrast, the level of ASPM was found to be unaltered upon overexpression or depletion of UBE3A in HEK293 cells in our study (Figures 5C and 6D). We suspect that the level of ASPM may be regulated by UBE3A in cell-cycle dependent, temporal-, spatial- or tissue-specific manner. For example, UBE3A interacts with HHR23A (human homologue of the yeast DNA repair protein Rad23) to degrade it specifically only in the S phase of cell cycle [39]. This possibility needs to be explored in future as we have observed that the levels of both ASPM and UBE3A decrease in S phase (Figure 5B). The likelihood of a proteasomal-independent function for the two proteins also exists.

The final number of neuronal progenitor cells determines the brain size, which results from the balance between proliferation and apoptosis [40]. The results from mouse Aspm siRNA study have shown that Aspm increases the number of asymmetric divisions in neuroepithelial cells leading to a reduced number of cells and as a consequence a smaller brain size [14]. However, an increased apoptotic mechanism seems to act in case of MCPH7 protein STIL to reduce the number of neuronal progenitors as mentioned above [27]. In the present study, we also observed an increased frequency of apoptotic cells in UBE3A knockdown clones (Figure 8B). This suggests that microcephaly may be caused by either a mechanism that affects cell fate determination and cell proliferation (as in the case of ASPM) or an increase in apoptotic signals in neural progenitor cells (as in the cases of STIL and UBE3A).

To summarize, the data presented here showed that the Angelman syndrome protein UBE3A interacts with primary microcephaly protein ASPM and, like ASPM and other MCPH proteins, localizes to centrosomes. We suggest that UBE3A is required for proper chromosome segregation and its loss-of-function leads to abnormal cytokinesis and apoptosis. It is possible that a similar defective chromosomal segregation mechanism is responsible for microcephaly in AS patients. We suggest that the identification of the pathway where ASPM and UBE3A intersect should now be the next most important goal.

## Materials and Methods

### Yeast two-hybrid (Y2H) analysis

For yeast two-hybrid analysis, we used the MATCHMAKER GAL4 Two-Hybrid System 3 (Clontech Laboratories) and *Saccharomyces cerevesiae* host strain AH109. The C-terminal region of ASPM (CTR) from amino acids 3,276–3,477 was used as a bait in yeast two-hybrid analysis (Figure 1A). To clone this region, total RNA was isolated from human fetal brain using TRI REAGENT™ (Sigma-Aldrich) according to the manufacturer's instruction. First-strand cDNA was synthesized from total RNA using the RevertAid™ H Minus First Strand cDNA synthesis kit (MBI Fermentas). RT-PCR primers used for the cloning of this region in-frame with the GAL4-DNA binding domain in the pGBKT7 vector are as follows: forward primer 5′-at*ggatcc*(*Bam* HI)acctagaggtagttactagattgtctc-3′ and reverse primer 5′-at*ctgcag*(*Pst* I)taaggaatgccaagcgtatccatcac-3′. RT-PCR product was first cloned in the TA cloning vector pTZ57R (MBI Fermentas). Following digestion of the TA clone with *Bam* HI and *Pst* I, the insert was eluted from the agarose gel and cloned in the pGBKT7 vector. The clone (pGBKT7-CTR) was sequenced on an ABIprism A310-automated sequencer to verify the sequence.

The pGBKT7-CTR clone was subsequently used as a bait in the yeast two-hybrid screening of a human fetal brain MATCMAKER cDNA library cloned in the activation domain vector pACT2 (Clontech Laboratories). The transformants obtained were screened for survival on plates with quadruple dropout medium (SD/-His/-Ade/-Leu/-Trp). The surviving transformants were further subjected to X-α-gal (5-Bromo-4-chloro-3-indolyl-α-D-galactopyranoside) plate assay for the detection of yeast α-galactosidase activity resulting from the expression of the *MEL1* reporter gene. The *MEL1* reporter gene is activated only when two proteins interact with each other in a GAL4 based system and the transformants appear blue in color. The plates were grown at 30°C in dark for 3 to 4 days and monitored for the appearance of blue color. pTD1 and pVA3 plasmids co-transformed in the yeast strain AH109 were used as a positive control for interaction. pVA3 encodes a fusion of the murine p53 protein (amino acids 72–390) and the GAL4 DNA-BD (amino acids 1–147). pTD1 encodes a fusion of the SV40 large T-antigen (amino acids 86–708) and the GAL4-AD (amino acids 768–881). p53 is known to interact with SV40 large T-antigen. A trasformant with pLAM5′ and pTD1 was used as a negative control. pLAM5′ encodes a fusion of the DNA-BD (amino acids 1–147) with human lamin C (amino acids 66–230). Lamin C and SV40 large T-antigen do not interact with each other. The surviving transformants were used for plasmid DNA isolation as described in the instruction manual for the MATCHMAKER GAL4 Two-Hybrid System 3. The insert in pACT2 plasmid was amplified using the following vector specific primers: forward 5′-ctattcgatgatgaagataccccaccccaccaaacc-3′, and reverse 5′-gtgaacttgcggggtttttcagtatctacga-3′. These primers amplify a 178 bp product when the pACT2 vector is devoid of any insert. The amplified insert was gel eluted and sequenced as described above. The BLAST analysis was subsequently performed to know the identity of the insert. The 714 bp fragment of *UBE3A* was recloned in the pACT2 vector (pACT2-UBE3A) as described above using cDNA from human fetal brain and following primers: forward primer 5′-taga*gaattc*(*Eco* RI)ccatggttgtctacaggaagctaatgg-3′, and reverse primer 5′-tcaa*ctcgag*(*Xho* I)ttacagcatgccaaatcctttggcata-3′. The pACT2-UBE3A and pGBKT7-CTR constructs were transformed again in yeast cells and tested for growth on a plate with quadruple dropout medium and X-α-gal with appropriate positive and negative controls.

### Antibody generation, Western blot analysis and co-immunoprecipitation

In order to generate an antibody against ASPM, a region from amino acids 544–1,059 (MTB) was cloned in the bacterial expression vector pET-33b(+) as described above using human fetal brain cDNA as a template. Following expression and purification of the bacterially expressed protein using a Ni^2+^-NTA column, it was injected in a rabbit. After four booster dosages, antiserum was collected and affinity-purified on a Protein A column (Bangalore Genei™). Pre-immune serum was also collected from the rabbit prior to injection of the bacterially expressed protein. Western hybridization was performed using a standard method and HRP-conjugated secondary antibodies (Bangalore Genei™), and an enhanced chemiluminescence detection kit (Millipore). Primary antibodies used for Western hybridization were as follows: raised anti-ASPM antibody (1∶1500 dilution), goat anti-ASPM (1∶200 dilution; sc-48883, Santa Cruz Biotechnology), goat anti-UBE3A antibody (1∶750 dilution; sc-8926, Santa Cruz Biotechnology), mouse anti-β-Actin (1∶20,000 dilution; A 5441, Sigma-Aldrich), mouse anti-HA tag (1∶5,000 dilution; H 9658, Sigma-Aldrich), and rabbit anti-Erk1/2 (1∶1,000 dilution; Cell Signaling).

For co-immunoprecipitation of endogenous ASPM and UBE3A, human fetal kidney tissue was lysed by homogenization in an ice-cold cell lysis buffer (Sigma-Aldrich) supplemented with a complete protease inhibitors mix (Sigma-Aldrich). The concentration of total protein was estimated by the Bradford assay. For equilibration, protein-A/G agarose beads (Sigma-Aldrich) were washed thrice in IP buffer containing detergent (20 mM HEPES pH 7.5, 150 mM NaCl, 1 mM EDTA, 0.5% NP-40, 1 mM sodium orthovanadate, 10 mM sodium fluoride, and 10% glycerol supplemented with a complete protease inhibitors mix) and then in IP buffer without detergent (20 mM HEPES pH 7.5, 150 mM NaCl, 1 mM EDTA, 1 mM sodium orthovanadate, 10 mM sodium fluoride, and 10% glycerol supplemented with a complete protease inhibitors mix). One mg of the lysate was pre-cleared by incubating it with normal IgG (pre-immune serum) and protein-A/G agarose beads. Pre-cleared lysate was incubated with an appropriate primary antibody for 3 hr at 4°C on a rotator. Thirty µl of the protein-A/G agarose beads were added to the lysate and primary antibody mix. The mix was incubated for 3 hr at 4°C on a rotator. Immune-complexes bound to protein-A/G agarose beads were pelleted down and washed thrice with an ice-cold IP buffer containing detergent and then with IP buffer without detergent. Finally, the protein-A/G beads were resuspended in 20 µl of 1 x SDS-gel loading buffer, boiled for 10 min and spun down by centrifugation at 12,000 rpm for 2 min at room temperature. The supernatant containing immunoprecipitates was then resolved on an appropriate percentage of SDS-PAGE and the signals were detected using Western hybridization as described above.

### Tissue samples

Human fetal tissue samples were obtained from a spontaneous abortus (18 weeks) which was frozen and stored at −80°C. Fetal tissue samples were collected at the Kempegowda Institute of Medical Sciences, Bangalore following approval from the Indian Institute of Science's ethics committee and written informed consent from the mother.

### Cell culture, transient transfection and cell cycle synchronization

HEK293, HeLa, HepG2 and A549 cells were cultured in a humidified 5% CO_2_ incubator at 37°C in DMEM supplemented with 10% (vol/vol) fetal bovine serum and 1x antimycotic/antibiotic mix (all from Sigma-Aldrich). Human cell lines were procured from the National Repository for cell lines at National Centre for Cell Sciences, Pune, India.

For overexpression of UBE3A, 2×10^6^ cells were transfected with an increasing concentration of the pCMV4-HA-UBE3A construct harbouring a full-length *UBE3A* gene (a kind gift from Prof. Ikuo Shoji, Kobe University, Japan). Transfection was carried out using Lipofectamine™ 2000 (Invitrogen) according to the manufacturer's instruction. For immunofluorescence analysis of HEK293 cells overexpressing HA-tagged UBE3A, two µg of pCMV4-HA-UBE3A was used for transfection.

To obtain cells synchronized at G1 phase, they were serum deprived in culture media for 72 hr. Cells were treated with 300 nM nocodazole (Sigma-Aldrich) for 15 hr to obtain M phase synchronized cells. To obtain cells synchronized in S phase, double thymidine treatment was used. Briefly, cells were treated with 2 mM thymidine (Sigma-Aldrich) for 18 hr. Cells were then washed, replaced with fresh medium and grown for 9 hr prior to a second treatment with 2 mM thymidine for 17 hr. DNA content was measured by FACS analysis on a flow cytometer (BD Caliber) and propidium iodide (PI) staining to confirm the cell cycle stage and the efficiency of synchronization.

### Immunofluorescence microscopy

HEK293 cells were grown on cover slips and allowed to adhere and gain morphology overnight. Cells were fixed with chilled methanol for 5 min. For α-tubulin staining, cells were treated with MTSB buffer (80 mM PIPES pH 6.8, 1 mM MgCl_2_ and 4 mM EGTA) for 1 min before fixing the cells. Cells were permeabilised and blocked for nonspecific sites with 2% bovine serum albumin in PBS plus 0.2% Triton-X-100. Cells were incubated with a primary antibody at 4°C for 5 hr, followed by 2 hr of incubation with a secondary antibody at room temperature. Primary antibodies used were as follows: raised anti-ASPM antibody 1∶15 dilution, goat anti-UBE3A antibody 1∶30 dilution (sc-8926; Santa Cruz Biotehnology), goat anti-UBE3A antibody 1∶10 dilution (sc-12380; Santa Cruz Biotechnology), mouse anti-γ-tubulin 1∶15 dilution (sc-17787; Santa Cruz Biotechnology), and mouse anti-α-tubulin 1∶750 dilution (T 5168; Sigma-Aldrich). Staining was visualized using an appropriate secondary antibody: mouse FITC (Sigma-Aldrich), rabbit TRITC (Sigma-Aldrich), rabbit Cy5 (Invitrogen), rabbit Cy2 (Molecular Probes), and goat Cy5 (Molecular Probes). Cells were stained with either 1 ug/ml Hoechst 33258 or 1 mg/ml propidium iodide (pseudo color blue) for 5 min. After washing, coverslips were mounted on slides with an antifade solution (Sigma-Aldrich) and photographed with a confocal Zeiss LSM 510 META microscope. The images were processed using LSM image browser (http://www.zeiss.co.uk/).

### shRNA knockdown

HEK293 cells were transfected with 1 µg of a shRNA construct directed specifically against UBE3A (HuSH 29mer shRNA; TR308522) purchased from OriGene Technologies. For transfection, Lipofectamine™ 2000 (Invitrogen) was used according to the manufacturer's instruction. Scrambled shRNA construct was transfected as a negative control. Puromycin resistant stable clones were picked up, expanded in culture, aliquoted and frozen in liquid nitrogen until use.

### *In vivo* detection of apoptosis

Caspase-3 activity was determined to estimate apotosis by flow cytometry (FACS Calibur, Becton Dickinson) using the CaspGLOW™ Fluorescein Active Caspase-3 Staining kit (BioVision). The kit provides sensitive detection of activated caspase-3 in living cells. It utilizes the caspase-3 inhibitor, DEVD-FMK, conjugated to FITC (FITC-DEVD-FMK) as a marker. For assay, 1×106 cells per well were seeded in a six-well plate, incubated for two days, and processed according to the manufacturer's protocol by adding FITC-DEVD-FMK. Cells were washed twice in wash buffer and resuspended in 300((l of wash buffer. Cells were then analyzed by flow cytometry using FL-1 channel (10,000 cells were measured for each sample).

## Supporting Information

Figure S1 **Validation of rabbit polyclonal anti-ASPM antibody raised in the present study by Western blot hybridization.** (A) Bacterially expressed and purified recombinant ASPM protein (MTB) probed with the raised anti-ASPM antibody. The quantity of recombinant protein (immunogen) loaded in the gel is shown. Note the anti-ASPM antibody recognizes the immunogen. (B) Western blot analysis of human fetal kidney lysate with the raised anti-ASPM antibody and a commercially available anti-ASPM antibody. Note both antibodies recognize the predicted 410 kDa band.
(TIF)
