# Supplementary figures and images for: Angelman Syndrome Protein UBE3A Interacts with Primary Microcephaly Protein ASPM, Localizes to Centrosomes and Regulates Chromosome Segregation
Source: PLoS One. 2011 May 25;6(5):e20397. doi: 10.1371/journal.pone.0020397 (PMC3102111; doi:10.1371/journal.pone.0020397)

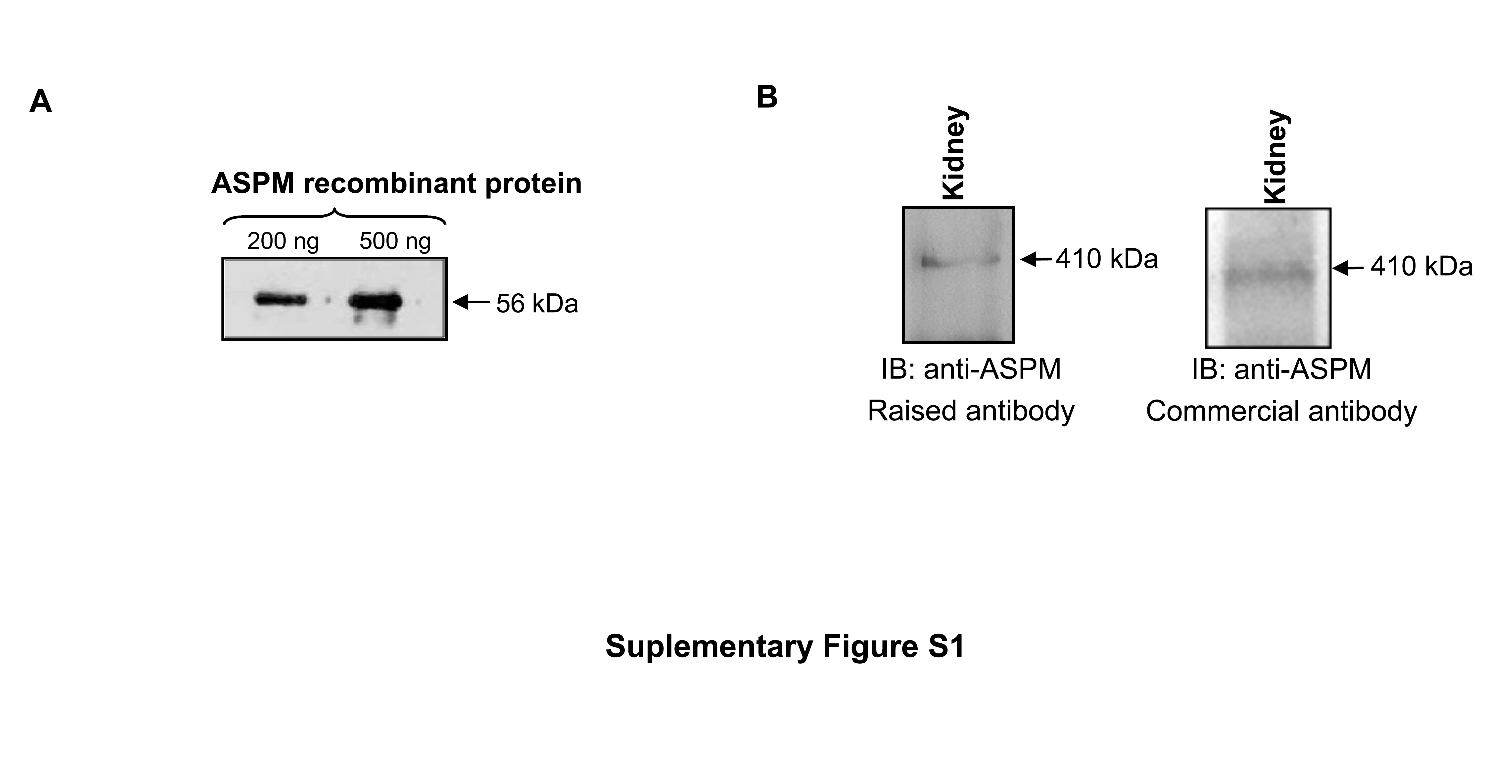

Supplement: Figure S1 — Validation of rabbit polyclonal anti-ASPM antibody raised in the present study by Western blot hybridization. (A) Bacterially expressed and purified recombinant ASPM protein (MTB) probed with the raised anti-ASPM antibody. The quantity of recombinant protein (immunogen) loaded in the gel is shown. Note the anti-ASPM antibody recognizes the immunogen. (B) Western blot analysis of human fetal kidney lysate with the raised anti-ASPM antibody and a commercially available anti-ASPM antibody. Note both antibodies recognize the predicted 410 kDa band. (TIF) [file pone.0020397.s001.tif]
